# Gleanings

**Published:** 1894-02

**Authors:** F. H. Lutze

**Affiliations:** Brooklyn, N. Y.


### GLEANINGS.

F. H. Lutze, M. D., Brooklyn, N. Y.

### Appetite, Taste, Hunger, and Thirst.

Appetite, loss of, after severe illness; does not return; no thirst. Antimon-crud.

Drink water, cannot, for the sight of it causes vomiting (during pregnancy). Bry.

Thirst constant for large quantities of ice-cold water, which is vomited so soon as it becomes warm in the stomach; mouth and tongue dry and burning; gurgling from stomach down, through abdomen, causing an involuntary stool from relaxed anus. Phos.

Appetite, great craving for food, in little children especially, but at sight of it this gives place to disgust. Sulph.

Aversion to fresh meat and potatoes. Thuja.

### Stomach.

Eructations tasting of food eaten twenty-four hours before. Bismuth-sub-nitr.

Burning in stomach, extending through to back. Bismuth, Carb-v., Con., Phos.

Hunger, anxiety, fright; felt in pit of stomach. Calc-c., Digit., Kali-c., Mezer., Phos.

Empty, all-gone feeling, as if bottom of stomach had dropped out. Puls., Thea.

Belching, followed by burning, soon after eating. Carbo-veg.

Gas accumulates in stomach excessively and belching. Carbol-ac.

Faint feeling in pit of stomach. Alumina, Baryta, Digit., Kali-c., Kalmia, Lobel., Oleand., Sepia.

Clawing in stomach and around the navel. Bell.

Gastralgia. Natr-m.

Emptiness and weakness in stomach; so great has to get up at night to eat, caused by mental emotion. Ignatia.

— and goneness at pit of stomach, even after eating, better by drinking brandy. Oleander.

Hunger, excessive at night; must eat to appease it. Carb-v.

Pain in stomach from jar of walking. Sepia.

— — — better from bending backward. Bism-sub-nit.

Swinging back and forth, sensation in stomach. Lycopod.

Sensation of something alive in the stomach. Crocus, Mancinell., Sang.

Stomach as if overloaded, until three hours after a meal. Amm-c.

Pain from stomach to spine, burning, causing a hot spot there in the spine; he bends head back to get relief. Bismuth.

Stomach, pain in, extends to back from 11 A. M. to 12 at night. Worse from touch and straightening out; better from pressure and drawing up the legs, and during and two hours after eating. Colocynth.

Stomach disordered by the mildest kind of food, flatulence incarcerated in stomach and whole lower abdomen ; great feebleness in stomach mornings. Kali-bich.

Emptiness, feeling of, in stomach, though want of appetite at dinner; a sensation of sinking in stomach before breakfast; patient wakes at night with great uneasiness in stomach, soreness and tenderness in a small spot to the left of the xiphoid appendage; sudden and violent pain in stomach in its anterior surface, a burning, contractive pain ; repletion after a mouthful of food. Kali-bich.

Cutting in stomach as with knives ; unable to digest potatoes or any starchy food. (There were no catarrhal symptoms of nose or chest, no thick, ropy mucous discharge.) Kali-bich.

Gastric symptoms worse P. M. Puls.

— — worse A. M. Sabad.

All-gone feeling in stomach, as if the bottom had dropped out. Puls.

### Hypochondres, Kidneys, Diaphragm.

Enlarged spleen. China, Nux-v., Sulph.

— — but no symptoms. Polymnia-urudalia (Bearsfoot).

Lying, when, on left side, pulling sensation in right hypochondrium. Worse lying on left side. Natr-sulph.

Numbness and tingling in whole left side; a distinct sensation in left wrist when bending the hand ; shooting pains about the heart, great anxiety and fear of an incurable heart disease ; tongue coated upon right side. Baptisia.

Sickening pain in left hypochondrium, going through to back, and a sensation as if a cord were drawn tight around the left side. Lachesis.

Stitches in region of liver, and tension across abdomen; worse evenings on lying down ; worse lying on right side or on painless side. Acon., Arn., Bry., Kali-c., Phos., Puls., Sepia.

### Abdomen.

Alive, sensation as if something were in abdomen. Calc-phos., Cann-s., Crocus, Cyclamen, Kali-iod., Nux-v., Silicea, Thuja.

Colic, periodical, several days in succession. Cedron, Chin-sulph., Colocynth, Lycopod., Kali-brom.

Dropsy of abdomen and limbs from anæmia. China.

Distention of abdomen. Bismuth, Caps., Cham., Colocynth., Iris-vers., Lil-tig., Natr-m., Plantago, Samb., Terebinth.

Falling, sensation of a heavy lump from navel to small of back. Laurocer.

— — — — hard body to right side on turning to that side, or to left side on turning to that. Lycopod.

Flatulence fetid. Æscul., Calc-phos., Diosc., Phos-ac.

Hold up, must, abdomen with hand, else cannot walk nor stand on her feet; bearing down in uterus and left ovary; pain comes in paroxysms, shooting down in left thigh. Lil-tig. (Staph., Agn.).

Pain, pinching, in abdomen; urinates very seldom; pain in bladder after urinating. Gamboge.

Weak abdomen, pressing down, must support it with hand. Agnus., Staph.

Swelling hard and red in left inguinal region, very painful; pain follows Poupart's ligament over top of hip bone to back and kidneys; sensation of tension on lifting. Argent-met.

Pain in small spot near umbilicus radiates in all directions; extends to stomach, liver, spleen, or uterus, testicles or spermatic cord, often jumps from place to place, even to distant parts. Dioscor.

Pressing or bearing down in abdomen. Anti-crud., Bell., Calc-c., Can., Caps., Lil-tig., Natr-c., Natr-m., Nitric-ac., Palad., Sep., Ustilag.

— — — must cross limbs. Sep.

— — — must press hand upon vulva. Lil-tig., Sep.

— — — must sit down. Lil-tig., Natr-m.

Pregnancy, during, aching pains in abdomen every night after going to bed; better getting up and moving about. Conium.

Colic, radiates in all directions from the umbilicus, like spokes of a wheel, even to toes; worse lying or bending double; better rising and walking. Dioscor. (Colocynth. the reverse.)

Cramps in abdomen, cholera-like, with diarrhœa and vomiting. Dulcam.

Weak and empty feeling in whole abdomen, with sensation of heat between the shoulder-blades. Phos.

Gurgling from stomach down through abdomen, causing an involuntary stool, from relaxed anus; desire for ice-cold water, which is vomited so soon as it becomes warm in stomach. Phos.

Great relaxation in abdomen after a loose stool, with burning desire to urinate, without much urine being passed. Phos.

